# Evolution of research trends and emerging hotspots in bioelectrical impedance analysis over the last two decades: a bibliometric analysis

**DOI:** 10.1080/15502783.2025.2523381

**Published:** 2025-06-26

**Authors:** Chaofeng Niu, Peiyu Zhang, Chao Zhang, Juwei Dong, Hao Liang, Di Xiao, Birong Liu, Lan Wei, Haixia Lai, Jiaqi Ye, Liyong Ma, Lijing Zhang

**Affiliations:** a Beijing University of Chinese Medicine, Department of Cardiology, Dongzhimen Hospital, Beijing, China; b Beijing University of Chinese Medicine, Department of Neurology, Dongzhimen Hospital, Beijing, China; c Beijing University of Chinese Medicine, Department of Acupuncture and Moxibustion, Dongzhimen Hospital, Beijing, China; d Hunan University of Chinese Medicine, Hunan Provincial Key Laboratory of TCM Diagnostics, Changsha, Hunan, China

**Keywords:** Research trends, hotspots, bioelectrical impedance analysis, body composition, nutrition, bibliometric analysis

## Abstract

**Background and objective:**

Over the last two decades, bioelectrical impedance analysis (BIA) has gained popularity as a method for assessing body compartments in nutrition studies, sports medicine, and evaluating hydration levels, fat mass, and fat-free mass variations in both healthy and diseased individuals. This study aims to offer researchers an overview of the research trends in BIA.

**Methods:**

The data was obtained from the Web of Science Core Collection database. Bibliometric analysis was conducted using a package of R software (Bibliometrix 4.0).

**Results:**

A total of 9471 articles have been published over the past 20 years, with an average annual growth rate of 10.1%. The research field primarily focuses on nutrition and dietetics, followed urology and nephrology, endocrinology and metabolism, general and internal medicine, engineering, geriatrics and gerontology, sport sciences, cardiovascular system and cardiology, physiology and science and technology-other topics. The research hotspots of BIA over the past 20 years have transitioned from “water” to “fat,” and subsequently to “sarcopenia.” “Sarcopenia” and “phase angle” (PhA) have emerged as recent research hotspots in the field of BIA.

**Conclusion:**

A total of 9471 articles have been published over the past 20 years, with an average annual growth rate of 10.1%. Nutrition and dietetics have consistently been the primary research areas in the field of BIA. “Sarcopenia” and “PhA” have emerged as recent research hotspots in the field of BIA. The application of BIA in clinical practice still holds significant untapped potential.

## 1. Introduction

Bioelectrical impedance analysis (BIA) is a method that evaluates body composition and tissue status by measuring the resistance of body cells and tissues to the flow of a radiofrequency alternating electrical current [1]. BIA is simple, fast, and noninvasive, which can be applied in various scenarios such as homes, hospitals, and health clubs [2]. Since the 1870s, Hermann [3] described the electrical properties of tissues, specifically noting that the conductive impedance of muscles and nerves is lower along the fiber direction compared to the vertical direction. These properties were further confirmed across a wider range of frequencies and on various types of tissues, including those that were damaged or underwent changes after death [4]. In 1907, Cremer [5] conducted electrical impedance measurements on isolated frog hearts, introducing the term “bioelectrical impedance analysis” for the first time. In 1937, Manx [6] reported on noninvasive measurements using skin electrodes on humans. He observed a correlation between the height of the pulse wave amplitude and the recorded curve of electrical impedance variation in the finger.

The applications of BIA have been developed over 60 years [7]. Siri [8] and Brozek et al. [9] proposed the two-compartment model encompassing fat mass (FM) and fat-free mass (FFM) to calculate body composition. FFM technically consists of all nonfat molecules, like water, protein, mineral, and residual. In 1962, Thomasett [10] pioneered the use of two frequencies bioimpedance analysis to estimate the total water volume of the human body. The technique of four-surface electrode BIA was initially introduced by Hoffer et al. [11] and Nyboer [12]. Hoffer [11] elucidated the correlation between total body impedance and total body water (TBW) content, with reference to tritium dilution techniques. Nyboer et al.‘s [13] research confirmed the association between changes in blood volume and alterations in body electrical impedance. In 1985, Lukaski et al. [14] and Segal et al. [15] initiated research using the first commercially available instrument. They measured whole-body resistance using a tetra-polar arrangement of electrodes, and FFM was determined by densitometric procedures. Since 1985, there has been an exponential growth in research related to impedance technology. In 1994, Piccoli et al. [16] introduced a novel method of bioelectrical impedance analysis, known as bioelectrical impedance vector analysis (BIVA). This approach enhances accuracy by removing the reliance on predictive equations. Through an empirical approach, BIVA enables direct analysis of the two components of the impedance vector (Z) – resistance (R, Ohm) and reactance (Xc, Ohm) – allowing for a semi-quantitative assessment of body composition, specifically in relation to body cell mass and hydration status. In 1996, the first position paper on BIA stated its usefulness in analyzing body composition for both healthy individuals and patients with chronic diseases. However, it acknowledged the influence of various factors (such as body position, hydration status, food consumption, ambient air and skin temperature, etc) and emphasized the need for further research to enhance the accuracy of body composition assessment [2]. In 2004, Kyle et al. [4,17] conducted a comprehensive review on the principles and clinical applications of BIA, providing valuable insights for improving its precision and applicability.

In the past 20 years, bioimpedance analysis has become an increasingly utilized method for estimating body compartments in nutrition studies, sports medicine, and assessing hydration rate, FM, and FFM differences across healthy and diseased populations [18]. However, there remains skepticism regarding the accuracy and clinical significance of BIA. BIA is a predictive method that simplifies and assumes based on population mean values, but it is generally considered applicable to all subjects with accuracy [19]. In addition, changes in the hydration of body tissues directly impact the results of BIA [20]. Zhu et al.‘s [21] study suggested that the continuous utilization of dynamic calf bioimpedance spectroscopy during dialysis was an accurate and precise method for estimating specific endpoints related to body hydration status. Hoyle et al. [22] found a strong correlation between BIA and deuterium oxide dilution in estimating TBW in elderly hyponatremic patients. Buffa et al. [23] proposed that BIVA was a promising tool for screening and monitoring the nutrition and hydration status in individuals with Alzheimer’s disease. Classic BIVA showed a strong correlation with body mass index, reflecting higher body mass in obese and athletic individuals. However, compared to BIA, it was less consistent, especially in diseased individuals. While classic BIVA struggled to detect changes in body fat percentage, specific BIVA gave more accurate results [24]. The specific BIVA introduced by Buffa et al. [25] in 2013 differs from classic BIVA by standardizing bioelectrical values for both height and transverse areas, rather than just height. Based on Ohm’s law, which states that resistance is proportional to length and inversely proportional to cross-sectional area, this approach aims to improve sensitivity to tissue properties and body composition by balancing the effects of body size and shape. Despite the numerous studies conducted on BIA in recent years, the progress in this field may have decelerated [19]. Our research aims to use bibliometric methods to visually and analytically examine the research on BIA conducted in the past 20 years. The objective is to provide researchers with an intuitive presentation of the research progress and changes in hot topics within the field of BIA.

## 2. Method

### 2.1. Search strategy

The data was obtained from the Web of Science Core Collection database. The search formula was as follows: ((TS=(bioelectrical impedance analysis)) OR TS=(bioimpedance analysis)) OR TS=(bioimpedance spectroscopy). All documents were exported as full record and cited references. A total of 9471 search results were exported (updated to 18 January 2024).

### 2.2. Bibliometric analysis

Bibliometric analysis was conducted using a package of R software (Bibliometrix 4.0) [26]. The built-in web program (Biblioshiny) in this package was used to perform preliminary analysis of the search results from the Web of Science Core Collection database. The analysis results were exported as an RData file for further examination and visualization using the R software.

## 3. Result

### 3.1. Descriptive bibliometric analysis

**Figure 1.**
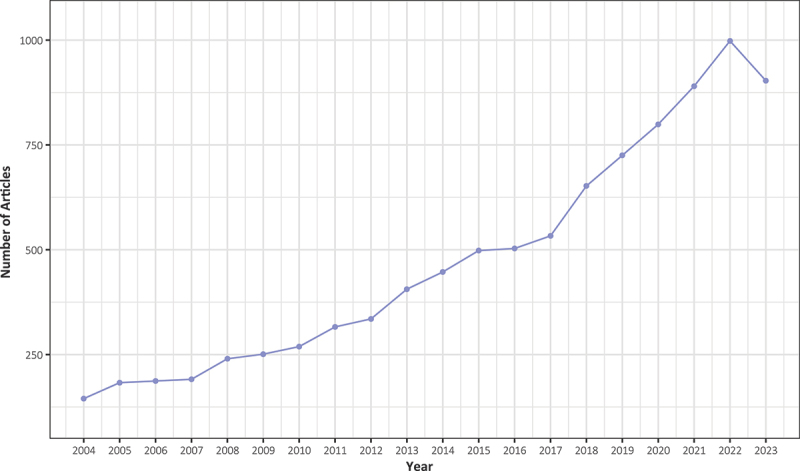
The scientific production of literature related to BIA spans from 2004 to 2023.

Figure 1 depicts the publication count of articles over the past 20 years. The annual publication volume has shown a consistent increase year by year. Table 1 presents the key information of 9471 papers published in the Web of Science Core Collection database from 2004 to 2023. The annual average growth rate of publication volume is 10.1%. The average number of citations per paper is 22.7. The articles are derived from a collection of 1685 sources. These articles collectively involve a total of 38,510 authors and encompass 11,285 author’s keywords.

**Table 1.** Main information.

Main information	Description	Value
Timespan	Years of publication	2004–2023
Documents	Total number of documents	9471
Annual growth rate (%)	The annual average growth rate of publication volume	10.1
Sources	The distribution frequency of different sources, such as journals, books, etc.	1685
Authors	Total number of authors	38510
Authors Appearances	The authors’ frequency distribution	64498
Authors of single-authored documents	The number of single authors per articles	143
Documents per Author	Average number of articles per author	0.246
Co-Authors per Documents	Average number of coauthors in each document	6.81
Author’s keywords	Total number of author’s keywords	11285
Keywords Plus	Total number of phrases that frequently appear in the title of an article’s references	9495
References	Total number of references	181393
Average citations per documents	Average number of citations in each document	22.7

### 3.2. Research field

**Figure 2.**
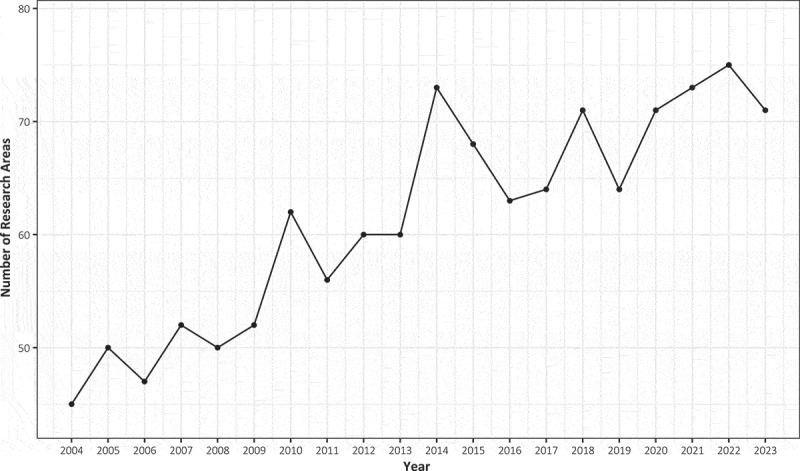
The research areas of literature related to BIA from 2004 to 2023.

**Figure 3.**
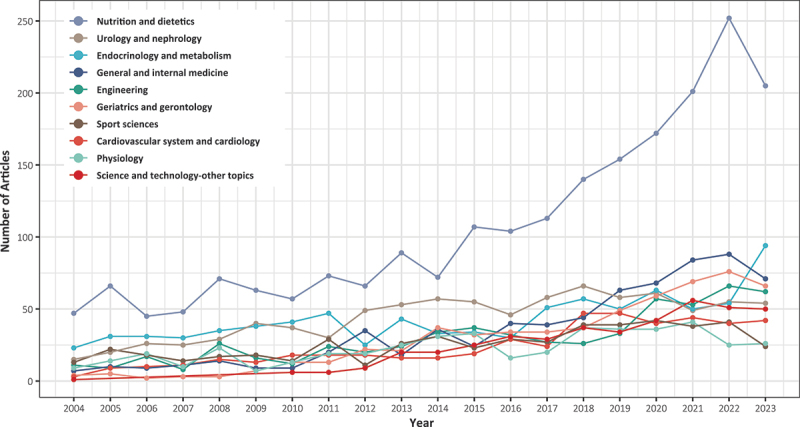
The trends in publications within the top ten research fields over the past 20 years.

Over the past two decades, Figure 2 illustrates a growing trend in research fields. Table 2 represents the top ten research areas with the highest number of publications: nutrition and dietetics (2145), urology and nephrology (883), endocrinology and metabolism (860), general and internal medicine (699), engineering (594), geriatrics and gerontology (586), sport sciences (515), cardiovascular system and cardiology (479), physiology (459) and science and technology-other topics (417). The top ten research fields accounted for approximately 80.6% of the total publications in the past two decades, with a combined number of 7637 articles. Figure 3 illustrates the fluctuations in the number of publications within the top ten research fields over the past 20 years. BIA has consistently been a research hotspot in the field of nutrition and dietetics.

**Table 2.** The number of articles in the top ten research fields with the highest publication count.

Research area	Number of articles
Nutrition and dietetics	2145
Urology and nephrology	883
Endocrinology and metabolism	860
General and internal medicine	699
Engineering	594
Geriatrics and gerontology	586
Sport sciences	515
Cardiovascular system and cardiology	479
Physiology	459
Science and technology-other topics	417

### 3.3. The most influential journals

**Figure 4.**
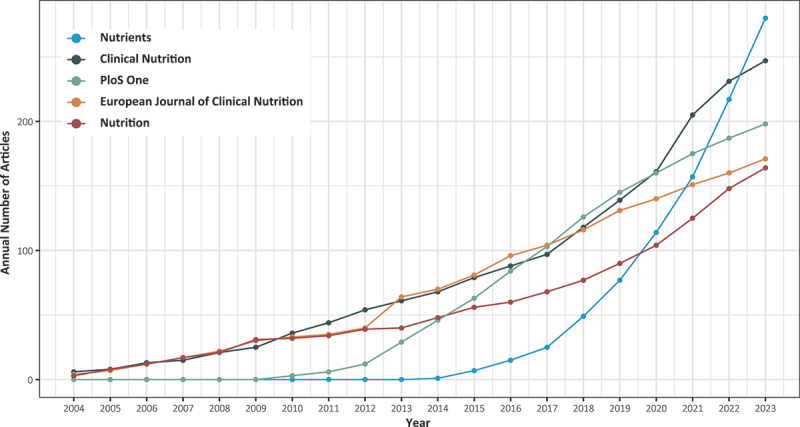
The top five journals in terms of publication volume and their publication trends over the past 20 years.

The most influential journals are Clinical Nutrition, European Journal of Clinical Nutrition, PloS One, American Journal of Clinical Nutrition, Nephrology Dialysis Transplantation, Nutrition, Obesity, Nutrients, International Journal of Obesity, and British Journal of Nutrition (Table 3). The top five journals, ranked by publication volume (Figure 4), are Nutrients (280), Clinical Nutrition (247), PloS One (198), European Journal of Clinical Nutrition (171) and Nutrition (164).

**Table 3.** The top ten influential journals.

Journal	IF	H Index	G Index	TC	NP
Clinical Nutrition	6.3	47	109	13127	247
European Journal of Clinical Nutrition	4.7	40	67	5542	171
PloS One	3.7	36	56	4575	198
American Journal of Clinical Nutrition	7.1	35	64	4943	64
Nephrology Dialysis Transplantation	6.1	34	58	3429	135
Nutrition	4.4	32	48	3225	164
Obesity	6.9	31	50	2588	60
Nutrients	5.9	30	47	3632	280
International Journal of Obesity	4.9	28	56	3158	72
British Journal of Nutrition	3.6	26	43	1955	67

IF, impact factor in 2023; TC, Web of Science Core Collection times cited count; NP, number of scientific productions.

### 3.4. The most influential articles

Table 4 displays the top ten articles with the highest number of citations. Within the top ten cited articles, four are from the field of geriatrics and gerontology, five are from the field of nutrition and dietetics, and one is from the field of environmental and occupational health. Nutrition and dietetics have consistently been the primary research areas in the field of BIA (Figure 3). Table 5 lists the top ten cited articles in the area of nutrition and dietetics.

**Table 4.** The top ten articles with the highest number of citations.

Paper	DOI	Year	Research Area	Times Cited
Cruz-Jentoft AJ, 2010, AGE AGEING	10.1093/ageing/afq034	2010	Geriatrics and gerontology	7788
Cruz-Jentoft AJ, 2019, AGE AGEING	10.1093/ageing/afy169	2019	Geriatrics and gerontology	5644
Chen LK, 2014, JOURNAL OF THE AMERICAN MEDICAL DIRECTORS ASSOCIATION	10.1016/j.jamda.2013.11.025	2014	Geriatrics and gerontology	2585
Chen LK, 2020, JOURNAL OF THE AMERICAN MEDICAL DIRECTORS ASSOCIATION	10.1016/j.jamda.2019.12.012	2020	Geriatrics and gerontology	2228
Kyle UG, 2004, CLINICAL NUTRITION	10.1016/j.clnu.2004.06.004	2004	Nutrition and dietetics	1770
Mourtzakis M, 2008, APPLIED PHYSIOLOGY, NUTRITION, AND METABOLISM	10.1139/H08–075	2008	Nutrition and dietetics	1441
Kyle UG, 2004, CLINICAL NUTRITION	10.1016/j.clnu.2004.09.012	2004	Nutrition and dietetics	1403
Cederholm T, 2017, CLINICAL NUTRITION	10.1016/j.clnu.2016.09.004	2017	Nutrition and dietetics	1160
Romero-Corral A, 2008, INTERNATIONAL JOURNAL OF OBESITY	10.1038/ijo.2008.11	2008	Nutrition and dietetics	921
Janssen I, 2004, AMERICAN JOURNAL OF EPIDEMIOLOGY	10.1093/aje/kwh058	2004	Environmental and occupational Health	779

DOI, Digital Object Identifier.

**Table 5.** The top ten cited articles in the area of nutrition and dietetics.

Paper	DOI	Year	Times Cited
Kyle UG, 2004, CLINICAL NUTRITION	10.1016/j.clnu.2004.06.004	2004	1770
Mourtzakis M, 2008, APPLIED PHYSIOLOGY, NUTRITION, AND METABOLISM	10.1139/H08–075	2008	1441
Kyle UG, 2004, CLINICAL NUTRITION	10.1016/j.clnu.2004.09.012	2004	1403
Cederholm T, 2017, CLINICAL NUTRITION	10.1016/j.clnu.2016.09.004	2017	1160
Romero-Corral A, 2008, INTERNATIONAL JOURNAL OF OBESITY	10.1038/ijo.2008.11	2008	921
Volkert D, 2019, CLINICAL NUTRITION	10.1016/j.clnu.2018.05.024	2019	614
Zamboni M, 2008, NUTRITION METABOLISM AND CARDIOVASCULAR DISEASES	10.1016/j.numecd.2007.10.002	2008	568
Norman K, 2012, CLINICAL NUTRITION	10.1016/j.clnu.2012.05.008	2012	560
Lee SY, 2008, CURRENT OPINION IN CLINICAL NUTRITION AND METABOLIC CARE	10.1097/MCO.0b013e32830b5f23	2008	428
Ling CHY, 2011, CLINICAL NUTRITION	10.1016/j.clnu.2011.04.001	2011	419

DOI, Digital Object Identifier.

### 3.5. Most influential authors

The top ten influential authors include Ward L. C., Pichard C., Heymsfield S. B., Norman K., Sardinha L. B., Silva A. M., Bosy-Westphal A., Davenport A., Gonzalez M. C. and Müller M. J. (Table 6)

**Table 6.** The top ten influential authors.

Author	H Index	G Index	TC	NP	Country
Ward L. C.	26	50	2749	92	Australia
Pichard C.	25	40	5374	40	Switzerland
Heymsfield S. B.	23	44	2871	44	USA
Norman K.	23	34	2563	34	Germany
Sardinha L. B.	23	32	1184	51	Portugal
Silva A. M.	23	34	1307	58	Portugal
Bosy-Westphal A.	22	30	3337	30	Germany
Davenport A.	22	38	1569	61	United Kingdom
Gonzalez M. C.	22	43	1892	43	Brazil
Müller M. J.	22	29	2619	29	Germany

TC, Web of Science Core Collection times cited count; NP, number of scientific productions.

### 3.6. Research countries and institutions

**Figure 5.**
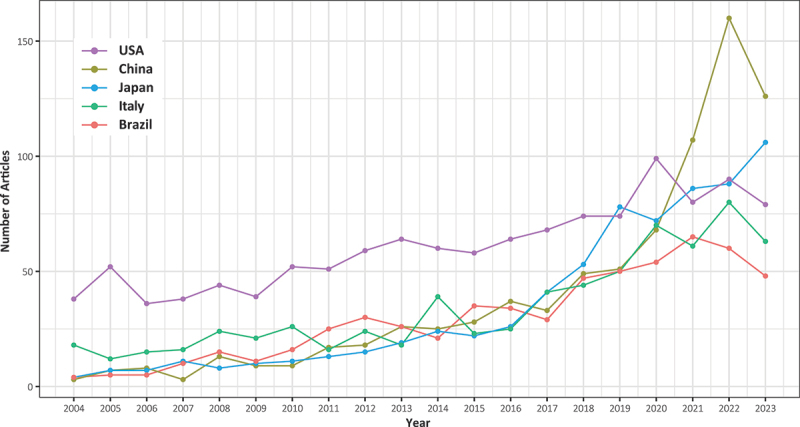
The top five countries in terms of publication volume and their trends in publications over the past 20 years.

**Figure 6.**
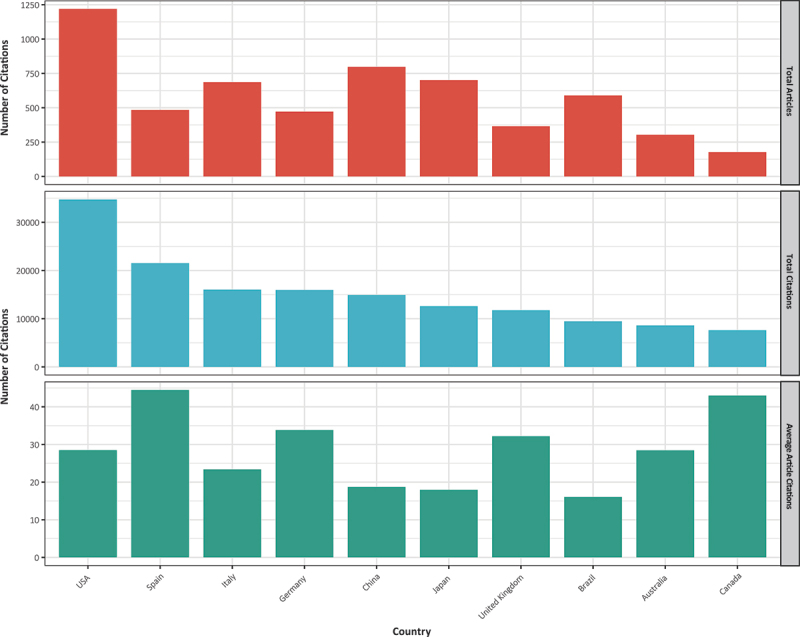
The comparison of the publication volume and citation count for the top ten countries.

The United States (1219), China (797), Japan (701), Italy (686), and Brazil (590) are the top five countries in terms of published articles in ascending order in the field of BIA. The number of publications in China has exhibited a pronounced upward trend over the past three years (Figure 5). We identified the top ten countries with the highest cumulative citation counts for published papers (Figure 6). The United States had the highest number of citations (34734), followed by Spain (21533), Italy (16041), Germany (15972), China (14913), Japan (12591), the United Kingdom (11749), Brazil (9475), Australia (8616), and Canada (7609).

**Figure 7.**
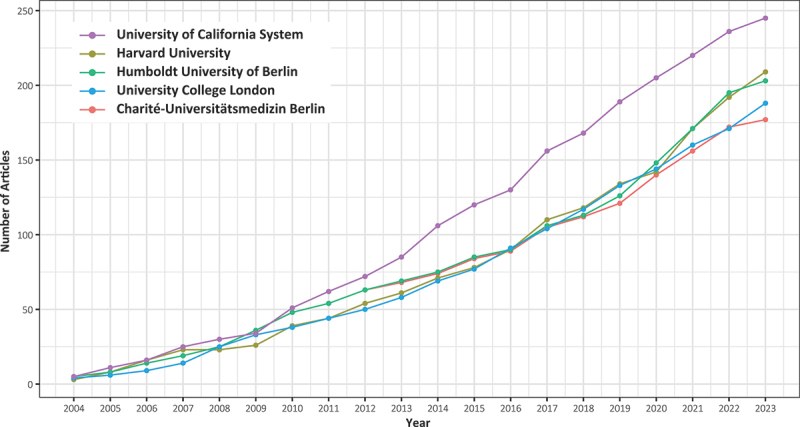
The top five institutions in terms of publication volume and their trends in publications over the past 20 years.

The top five institutions (Figure 7), in terms of the highest number of publications, are University of California System (245), Harvard University (209), Humboldt University of Berlin (203), University College London (188) and Charité-Universitätsmedizin Berlin (177).

### 3.7. Research hotspots analysis

**Figure 8.**
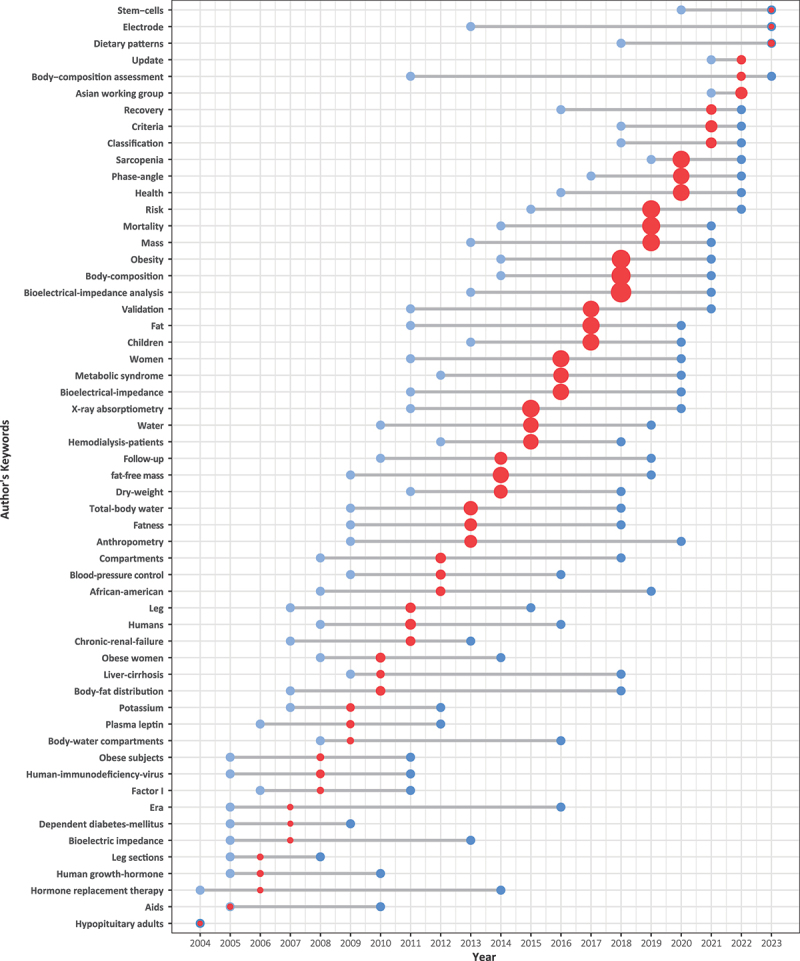
The temporal dynamics of the author’s keywords.

In our study, we identified a total of 11,285 author’s keywords in 9471 articles published from 2004 to 2023. Figure 8 illustrates the temporal dynamics of the author’s keywords. Each keyword is represented by three points, corresponding to the first quantile, median, and third quantile of the publication year from left to right. The size of the points reflects the number of published papers. The top ten author’s keywords with the highest frequency of occurrence include body composition (1643), sarcopenia (760), obesity (737), bioelectrical impedance analysis (715), bioimpedance (632), phase angle (PhA, 409), bioelectrical impedance (355), malnutrition (300), hemodialysis (275) and fat mass (273).

For an author’s keyword, the proximity of its dark blue dot to the right and the size of its red dot in the middle of the graph indicate the relative recency of publications associated with this keyword and the volume of papers which have been published. Figure 8 indicates that the research hotspots of BIA over the past 20 years have transitioned from “water” to “fat,” and subsequently to “sarcopenia.” “Sarcopenia” and “PhA” have emerged as recent research hotspots in the field of BIA.

## 4. Discussion

The literature pertaining to BIA has exhibited a remarkable surge in recent years, indicative of exponential growth. Furthermore, the research domain continues to expand steadily. We conducted a comprehensive bibliometric review of BIA research from 2004 to 2023. A total of 9471 articles have been published over the past 20 years, with an average annual growth rate of 10.1%. The research field primarily focuses on nutrition and dietetics, followed by urology and nephrology, endocrinology and metabolism, general and internal medicine, engineering, geriatrics and gerontology, sport sciences, cardiovascular system and cardiology, physiology and science and technology-other topics.

### 4.1. The most influential articles

The first and second most cited articles pertain to the definition and diagnostic consensus of sarcopenia in Europe, published in 2010 [27] and 2019 [28], respectively. The articles mentioned the utilization of BIA as a means to assess skeletal muscle mass index (SMI), serving as a diagnostic criterion for sarcopenia. While computed tomography (CT) and magnetic resonance imaging (MRI) remain the gold standards for studying muscle mass, BIA can serve as a portable alternative for measuring muscle mass [27]. The third and fourth most cited articles are the consensuses led by Prof. Liang-Kung Chen and his colleagues [29,30] on sarcopenia in Asia. The consensuses discussed the utilization of BIA in measuring SMI specifically for Asian populations. Although BIA’s accuracy in sarcopenia diagnosis has been validated, it heavily relies on equipment equations and assessment conditions, such as temperature, humidity, and skin condition [29]. The fifth and seventh most cited articles are reviews authored by Prof. Ursula G. Kyle and her colleagues, focusing on BIA. These two reviews are considered pivotal articles in the field of BIA in recent years, providing a detailed expound on the principles, methods, and clinical applications of this technique [4,17]. The sixth most cited article is a study conducted by Prof. Marina Mourtzakis and her colleagues, published in 2008. The study aimed to compare body composition measurements of cancer patients using CT, dual-energy X-ray absorptiometry (DXA), and BIA methods. The findings indicated that BIA, when compared to DXA and CT, demonstrated significant discrepancies in estimating FFM, resulting in either overestimation or underestimation [31]. The eighth most highly cited article refers to the European Society for Clinical Nutrition and Metabolism (ESPEN)‘s publication of clinical nutrition definition and terminology guidelines in 2017 [32]. The article highlights that BIA is a fast and noninvasive technique used to estimate body composition. However, it requires strict adherence to protocols, including a minimum 2-hour fasting period and voiding before the test. Single-frequency BIA is a widely used method that employs a validated formula to estimate TBW and FFM. On the other hand, multi-frequency BIA (MF-BIA) and bioelectrical impedance spectroscopy (BIS) are capable of calculating intracellular water (ICW), extracellular water (ECW), TBW, and FFM. BIS provides insights into the distribution of ICW and ECW, enabling the prediction of FFM. Furthermore, the BIA-derived PhA exhibits a robust prognostic value [32,33]. The ninth most cited article is a 2008 study by Dr. Abel Romero-Corral et al. [34], which examines the accuracy of body mass index (BMI) in diagnosing obesity. Through a comparison of BMI with body fat percentage measured by BIA, the study revealed limitations in using BMI as a diagnostic tool for obesity, as it cannot differentiate between body fat percentage and lean mass. However, by utilizing BIA, individuals with moderate BMI can be further categorized based on their measured body fat levels. The tenth most cited article is a study conducted by Prof. Ian Janssen and published in 2004. The study focused on using BIA to measure body composition and predicting physical disability based on SMI [35]. From highly cited articles, the research hotspots of BIA in recent years have mainly focused on sarcopenia and obesity. These studies have benefited from the advancements in body composition analysis applications of BIA.

In the realm of nutrition and dietetics research, it is noteworthy that the top five highly cited articles also rank among the top ten most cited articles. The sixth most highly cited article in this field pertains to ESPEN’s clinical nutrition and hydration guidelines for geriatrics, which were published in 2019. This guideline emphasized that BIA should not be employed as a diagnostic method for evaluating the hydration status of elderly individuals, as its efficacy had not been sufficiently established [36,37]. The seventh most cited article is a review authored by Prof. Mauro Zamboni and his colleagues, discussing sarcopenic obesity. This review mentioned the use of BIA in diagnosing this novel form of obesity [38]. The eighth most cited article is also a review authored by Prof. Kristina Norman and her colleagues, which focuses on the analysis of bioelectrical PhA and impedance vector. She suggests that the bioelectrical PhA is a valuable prognostic marker and should be used as a screening tool to identify patients at risk of impaired nutritional and functional status. Additionally, BIVA can be employed for nutritional assessment and monitoring when body composition calculations are not possible [39]. The ninth most cited article is a review by Prof. Seon Yeong Lee, which explores different methods for analyzing body composition and provides a comprehensive comparison among them. The main limitation of BIA or BIS highlighted in this review is its relatively lower accuracy [40]. The tenth most cited article is a study conducted by Prof. Carolina H.Y. Ling. This study discovered that direct segmental MF-BIA was an effective tool for evaluating whole body composition and segmental lean mass measurement in middle-aged populations when validated against DXA [41]. In recent years, BIA research has primarily concentrated on enhancing the clinical applicability of body composition accuracy through the development of various novel technologies. These advancements have been prominently featured in highly cited articles within the most prolific research field.

### 4.2. Most influential authors

In our study, the H-index of the authors was not their actual H-index, but rather a calculated value derived from the bibliometrix package using literature search in the field of BIA.

Prof. Leigh C. Ward is affiliated with the School of Chemistry and Molecular Biosciences at the University of Queensland, Australia. He has made noteworthy contributions to the field of nutrition, particularly in the area of protein metabolism. Starting in 1992, Prof. Ward [42,43] progressively shifted his research focus toward the utilization of BIA for predicting human body moisture content. For almost three decades following that, he dedicated himself to researching the application of BIA in measuring human body composition. Prof. Claude Pichard holds a distinguished position as a nutrition professor at the Geneva University Hospital in Switzerland. His area of expertise lies in conducting research focused on patient nutrition management. During the 1990s, Prof. Pichard’s [44,45] research primarily centered around the analysis methods for human body composition, with a particular emphasis on DXA. However, starting from around 2000, his focus gradually shifted toward BIA as an alternative method for assessing body composition [46,47]. Prof. Steven B. Heymsfield is affiliated with the Pennington Biomedical Research Center in the United States. His research primarily revolves around human obesity, with a particular emphasis on topics such as energy balance regulation, weight loss treatments, the impact of co-morbidities, and the creation of mathematical models related to these areas. Additionally, he has a longstanding interest in advancing the field of body composition evaluation by exploring innovative techniques like 3D optical imaging, functional MRI, and positron emission computed tomography, and their application in studying human metabolism. Prof. Heymsfield [48] has been devoted to the investigation of human body composition measurement since the 1980s. In the 1990s, he progressively shifted his focus toward the application of BIA for body composition analysis. He has made significant contributions to the field by conducting comprehensive research on the methodologies involved in accurately determining skeletal muscle mass using BIA [49,50]. Prof. Kristina Norman is affiliated with the German Institute of Human Nutrition and Charité Universitätsmedizin Berlin. Her research focuses on age-related changes in nutritional physiology, malnutrition, frailty, and sarcopenia in advanced-age clinical and community-dwelling populations. Since 2007, she has been actively engaged in research related to BIA, with a primary emphasis on monitoring the nutrition and functional status of the elderly individuals [51]. She advocates for the consideration of bioimpedance PhA as a screening tool to identify high-risk patients with impaired nutritional and functional status [39,52]. Prof. Luis Bettencourt Sardinha is affiliated with the University of Lisbon. The focus of his research has been on advancing the methods used to assess human body composition, as well as investigating the graded and dose-response relationships between sedentary behavior, physical activity, and fitness in relation to physiological attributes throughout the lifespan [53,54]. Over the past two decades, Prof. Sardinha has focused on the accuracy of various methods used in the analysis of human body composition. His investigations have encompassed techniques such as BIA, DXA, and air displacement plethysmography [53,55]. Prof. Analiza Mónica Silva is also affiliated with the University of Lisbon. For nearly two decades, she has been devoted to conducting research on models for body composition analysis. Her body composition analysis model prediction primarily focuses on athletes as the primary research population [56,57]. In 2023, she and a multitude of experts in the field advocated for the establishment and enhancement of the BIA international database [58]. Prof. Anja Bosy-Westphal is affiliated with the Faculty of Agricultural and Nutritional Sciences at Christian Albrechts University, Kiel, Germany. She has collaborated extensively with Prof. Manfred James Müller. She has conducted extensive research in the field of nutrition and metabolism. Her research on BIA began around 2005 [59]. For the past two decades, she has been devoted to investigating the precision of measuring human body composition using BIA and examining the correlation between human body composition and metabolic status [60,61]. Honorary Prof. Andrew Davenport [62], affiliated with the University College London, Department of Renal Medicine, has been actively engaged in research since 2009 focusing on the utilization of BIA for evaluating the fluid status of patients undergoing dialysis. His study showed that MF-BIA held promise as a valuable tool for assessing the nutritional status of peritoneal dialysis patients [63]. Serial estimations using MF-BIA have the potential to identify short-term changes in body composition, aiding in the recognition of dynamic variations over time [64,65]. Prof. Maria Cristina Gonzalez is affiliated with Universidade Federal de Pelotas. She has conducted postdoctoral research at the Pennington Biomedical Research Center in the United States. Her research focuses on body composition analysis and nutritional assessment. She believes that adherence to principles such as using correct terminology, selecting appropriate equations and cutoffs, and employing proper statistical approaches is crucial in all BIA studies [66]. She has collaborated extensively with Prof. Heymsfield [67,68] on research focused on using BIA for the assessment of obesity and sarcopenia. Prof. Manfred James Müller has previously held the position of director at the Institute of Human Nutrition and Food Science at Christian Albrechts University, Kiel, Germany. He has made notable contributions to the field of childhood obesity [69]. Since 2005, he has been actively involved in researching the measurement of body FM using bioelectrical impedance [59]. His research findings indicated that, at the population level, measuring body FM did not provide any advantage over using BMI and waist circumference in predicting obesity-related metabolic risks. However, considering both protective components of body composition such as FFM or muscle mass, along with risk-associated components like visceral or abdominal FM, may lead to an improved approach for assessing metabolic risks associated with obesity [60].

In addition, two experts in nephrology have also conducted extensive research on the application of BIA. Prof. Adrian Covic is an expert in the field of nephrology and internal medicine, currently serving as a professor at Grigore T. Popa University of Medicine and Pharmacy in Romania. His research focuses primarily on chronic kidney failure and dialysis. Since 2011, Prof. Covic [70] has been actively involved in researching the application of BIA in patients undergoing blood filtration procedures, particularly focusing on evaluating the hydration status of dialysis patients. His primary objective is to enhance fluid management and blood pressure control by utilizing BIA as a tool for assessing the hydration levels of individuals receiving dialysis treatment [71,72]. Prof. Nathan W. Levin is affiliated with the Renal Research Institute in New York and Icahn School of Medicine at Mount Sinai. He has conducted extensive research focusing on fluid management for patients undergoing dialysis. Starting from approximately 1995, his focus shifted toward exploring the utilization of BIA for evaluating the hydration status of dialysis patients [73]. According to his belief, segmental BIS facilitates a more accurate estimation of tissue masses and fluid volumes by virtue of the reduced electrode distance and the inherent cylindrical shape of the segments [74].

### 4.3. Research hotspots

Based on the analysis of highly cited articles and influential authors, it is evident that in recent years, the primary clinical applications of BIA have been focused on the field of sarcopenia. According to the 2014 consensus report of the Asian Working Group for Sarcopenia, BIA is endorsed for the diagnosis and assessment of intervention plans in sarcopenia [29]. The European consensus on the definition and diagnosis of sarcopenia in 2010 also acknowledged that BIA is a straightforward and effective method for estimating skeletal muscle mass, potentially replacing DXA. The consensus report provides a comprehensive list of the measurable variables and cutoff points of BIA as a diagnostic tool for sarcopenia [27]. BIA, as a measurement tool for sarcopenia, derives benefits from its ability to perform body composition analysis. Figure 8 indicates that the research hotspots of BIA over the past 20 years have transitioned from “water” to “fat,” and subsequently to “sarcopenia.” This evolution reflects the changing focus of researchers on the measurement variables of body composition analysis in BIA. Initially, the primary application of BIA for body composition analysis was centered around fluid management in patients [42]. However, in recent years, it has been increasingly utilized to assess the nutritional status of patients. Consequently, highly influential researchers in this field are predominantly experts from the field of nutrition.

BIA has become increasingly popular as a method for assessing body composition and health status in children and adolescents [75]. Talma et al. [76] suggested that BIA was a practical method for estimating percentage body fat in children and adolescents. However, its validity and measurement accuracy were found to be unsatisfactory. One limitation of BIA, particularly regarding the selection of predictive equations, is still under active development. Alissa et al. [77] recommended utilizing the Hamilton equation for predicting body composition values in adolescents with severe obesity. However, a single equation cannot fully account for variations in age, race, gender, and body composition, so predictive equations must be chosen carefully. Almeida et al.‘s [78] study demonstrated that BIVA can be used to assess body compartments in children and adolescents. BIVA improves accuracy by eliminating the need for predictive equations. This method effectively monitored lean and fat mass as well as hydration during development and maturation. It was also suitable for identifying cardiovascular risk in these age groups, though it was not able to stratify the population into different nutritional categories as the BMI/age curves do. Many studies also demonstrated that BIA could be used to estimate bone mineral content. However, compared to DXA, BIA often overestimated bone mineral content [79] and was inaccurate in assessing the bone mineral content in healthy children [80].

In the field of sports science, BIA also has a wide range of applications. Di Credico et al.‘s [81] study provided BIA data for elite young male and female handball players, which could help identify key bioelectrical impedance vector regions. This information allowed trainers to design targeted training programs to promote appropriate vector changes. Castizo-Olier et al. [82] suggested that BIVA was a relatively new technique with potential in sport and exercise, especially for assessing soft-tissue injuries.

Electrical bioimpedance measurements can be conducted using a single frequency, frequency sweeps, or through time-domain analysis. The electrical bioimpedance of materials such as tissue, organs, or human body parts is represented in raw form, including magnitude and PhA, real and imaginary components, resistance and reactance, or dielectric parameters like complex permittivity or conductivity [1]. The PhA, a parameter in BIA, represents the phase shift or delay between the applied voltage and the measured current, and serves as an indicator of cellular membrane integrity. Consequently, PhA is also considered a marker of fluid distribution, particularly ICW, which affects ECW and TBW [83,84]. PhA increases with age, particularly after puberty, while changes in younger subjects are less defined. A clear sex difference was observed in adolescents, likely due to puberty. Limited evidence suggests that PhA also rises in individuals with very high BMI [85]. In recent years, there has been increasing attention on the relationship between PhA and diseases. Gupta et al.’s [86] study showed that bioelectrical impedance PhA serves as a prognostic indicator for advanced cancer patients. Salazar et al. [87] found that PhA spectroscopy has the capability to differentiate between healthy and scar myocardium. Gulin et al. [88] found that lower PhAs in gastrointestinal cancer patients predict a higher risk of postoperative complications, indicating PhA as a prognostic indicator for surgical outcomes. Norman [39,52] believed that PhA had the potential to be utilized as a screening tool for identifying malnutrition conditions. Akamatsu et al.‘s [89] study demonstrated that PhA reflected muscle quality and accurately detected sarcopenia, highlighting its potential as a simple and effective index for assessing muscle quality, which could enhance sarcopenia diagnosis. A meta-analysis showed that PhA could serve as a surrogate marker for muscle quality, particularly reflecting muscle composition. Future studies should use BIA with standardized protocols to establish PhA cutoff values, improving its diagnostic accuracy and clinical applicability [90]. Simultaneously, research on composite impedance metric is progressively broadening the application range of BIA [91]. The application of BIA in clinical practice still holds significant untapped potential.

### 4.4. Limitation

The current bibliometric segmentation algorithms are lacking in intelligence and accuracy when it comes to extracting specific keywords. Therefore, future bibliometric research should focus on enhancing the semantic understanding of citation data. This improvement will enhance the accuracy of segmentation statistics and enable a more precise and intelligent extraction of bibliometric knowledge.

## 5. Conclusion

A total of 9471 articles have been published over the past 20 years, with an average annual growth rate of 10.1%. Nutrition and dietetics have consistently been the primary research areas in the field of BIA. “Sarcopenia” and “PhA” have emerged as recent research hotspots in the field of BIA. The application of BIA in clinical practice still holds significant untapped potential.

## Supplementary Material

Supplemental Material

## Data Availability

All data generated or analyzed during this study are included in this article and its supplementary material files. Further enquiries can be directed to the corresponding author.

## Abbreviations

BIA: Bioelectrical impedance analysis
BIS: Bioelectrical impedance analysis
BIVA: Bioelectrical impedance vector analysis
BMI: Body mass index
CT: Computed tomography
DXA: Dual-energy X-ray absorptiometry
ECW: Extracellular water
ESPEN: The European Society for Clinical Nutrition and Metabolism
FFM: Fat-free mass
FM: Fat mass
ICW: Intracellular water
MF-BIA: Multi-frequency bioelectrical impedance analysis
MRI: Magnetic resonance imaging
PhA: Phase angle
SMI: Skeletal muscle mass index
TBW: Total body water
